# The Probiotic Strain Hafnia Alvei HA4597 and Its Protein Extract Selectively Activate Membrane Digestive Enzymes of Intestinal Cells in Mice

**DOI:** 10.3390/life16091445

**Published:** 2026-08-30

**Authors:** Anastasiya L. Katargina, Lyudmila V. Gromova, Alexander S. Polozov, Nadezhda M. Grefner, Vasily A. Zolotarev, Julia V. Dmitrieva, Catherine A. Lukina, Elena A. Vershinina, Alexander N. Suvorov, Nina S. Pestereva, Sergueï O. Fetissov

**Affiliations:** 1 Pavlov Institute of Physiology, Russian Academy of Sciences, 199034 Saint-Petersburg, Russia; seppal@infran.ru (A.L.K.); zolotarevva@infran.ru (V.A.Z.); dmitrievayv@infran.ru (J.V.D.); lukinaea@infran.ru (C.A.L.); ver_elen@mail.ru (E.A.V.); 2 Sechenov Institute of Evolutionary Physiology and Biochemistry, Russian Academy of Sciences, 194223 Saint-Petersburg, Russia; polozovalexandr20@gmail.com; 3 Institute of Experimental Medicine, 197376 Saint-Petersburg, Russia; ngrefner@yandex.ru (N.M.G.); alexander_suvorov1@hotmail.com (A.N.S.); pesterevans@yandex.ru (N.S.P.); 4 Neuroendocrine, Endocrine and Germinal Differentiation and Communication Laboratory, Inserm UMR1239, University of Rouen Normandie, 76130 Mont-Saint-Aignan, France

**Keywords:** *Hafnia alvei*, probiotics, intestinal digestive enzymes, defense systems of the small intestine

## Abstract

Oral supplementation of the *Hafnia alvei* (*H. alvei*) HA4597 probiotic strain has been shown to reduce appetite, body weight and fat content in overweight humans and obese mice. However, it remains unknown if *H. alvei* may also influence the gut digestive function. Thus, in the present study, the structural parameters of the intestine and the activity of digestive enzymes of the brush border enterocytes were analyzed in mice after they received a suspension of live *H. alvei* bacteria or their total protein extract for 16 days. Using electron microscopy, bacteria resembling *H. alvei* were found on the surface of microvilli of colonic enterocytes of mice treated with *H. alvei* suspension. In the group receiving *H. alvei* protein, there was a trend toward an increase in the number of mucus-producing goblet cells on the villi of the jejunum, and in both groups receiving *H. alvei* preparations, the number of intraepithelial lymphocytes on the villi of the ileum increased (in the group receiving *H. alvei* protein, there was a tendency towards an increase; in the group receiving *H. alvei* suspension, there was a significant increase). Treatment with both *H. alvei* preparations led to an increased activity of alkaline phosphatase in the chyme of the duodenum and jejunum and also in the jejunal mucosa, correlating negatively with visceral body fat content. The use of both *H. alvei* preparations had no significant effect on the activity of glucoamylase, maltase and aminopeptidase N in the intestinal mucosa. Thus, the 16-day administration of *H. alvei* bacteria or their protein extract increases the protective properties of the intestine, including the mucous barrier. Moreover, increased activity of alkaline phosphatase, a fat absorption-limiting enzyme, may contribute to the known anti-obesity effects of *H. alvei*. Taken together, *H. alvei* preparations display beneficial effects on gut health.

## 1. Introduction

The prevalence of obesity and diabetes in the world has been noticeably progressing in recent decades. Many studies suggest that dysbiotic changes in the gut microbiota are involved in the initiation and progression of these metabolic disorders [1,2]. In this regard, strategies aimed at modulating the gut microbiota composition, including the use of probiotics, prebiotics, or fecal microbiota transplantation, are promising new approaches for the treatment of obesity and diabetes. Several studies using traditional probiotics based on various *Lactobacillus* and *Bifidobacterium* species or their combinations have shown positive effects in obesity and diabetes by improving lipid metabolisms and reducing inflammation without significant effect on appetite [3,4,5].

In recent years, a new research effort led to the development of a new generation of probiotics capable of reducing appetite and body weight. As such, a strain of *Hafnia alvei* (*H. alvei*) HA4597 bacteria, which can be present in gut microbiota of healthy humans and are used for the production of some types of soft cheeses, was identified and validated as a probiotic to control appetite and body weight [6]. The selection of *H. alvei* was based on its production of the caseinolytic protease B (ClpB) protein displaying anorexigenic and weight-reducing properties in obese mice [6]. In fact, earlier, enterobacterial ClpB was identified as a conformational antigen mimetic of α-melanocyte-stimulating hormone (α-MSH), a key anorexigenic and catabolic regulatory peptide [7]. Increased production of ClpB by gut bacteria was associated with a satietogenic effect perceived by the central nervous system [8]. Recently, the ability of ClpB to directly activate melanocortin type 4 receptor was demonstrated by an in vitro study [9]. The efficacy of the *H. alvei* HA4597 strain in reducing body weight and fat content as well as increasing the feeling of satiety has been shown in overweight humans undergoing a moderate hypocaloric diet [10].

Thus, while the current use of *H. alvei*-based probiotic is based on its known mechanism of action involving the host melanocortin system signaling satiety, it has yet to be determined whether *H. alvei* may also influence intestinal digestive and protective functions, thereby contributing to health beneficial effects. To respond to these questions, in the present study in healthy mice we analyzed the effects of 16-day administration of *H. alvei* bacteria and their total protein extract on the structural parameters of the intestine and functional activity of enterocyte brush border enzymes involved in the hydrolysis of carbohydrates (glucoamylase, maltase), proteins (aminopeptidase N, APN) and fats (alkaline phosphatase, ALP). In addition to their key role in digestion, APN is also involved in cholesterol absorption and immune responses [11,12], and ALP is involved in fat absorption and antigen defense [12,13,14,15]. The presence of *H. alvei* near the microvilli of colonic enterocytes was analyzed using electron microscopy.

## 2. Materials and Methods

### 2.1. Animals

The study was performed on 7–8-month-old C57BL/6J male mice, which were descendants of a breeding core purchased from Jackson Laboratory (Bar Harbor, ME, USA). The strain is maintained in the Biocollection of the I.P. Pavlov Institute of Physiology, Russian Academy of Sciences. The study was conducted in accordance with the Declaration of Helsinki and the recommendations National Institutes of Health and the European Community Council for the Care and Use of Laboratory Animals and approved by the Animal Care and Use Committee at the Pavlov Institute of Physiology (Animal Welfare Assurance #A5952-01), approval No. 03/15 dated 15 March 2022.

Mice were kept in a vivarium under conditions of artificial light (12/12 h, switching on at 8:00) and a constant temperature (23 ± 1 °C), 4–5 mouse per cage, with free access to water and food. The standard granulated food PK-120 was used as a diet, which contained 67% carbohydrates, 19% proteins and 5% fats with an energy value of 2.99 kcal/g (“Mest” LLC, Moscow, Russia). Animals were placed in individual cages 10 days prior to the start of the experiments.

### 2.2. Bacterial Culture

Live *Hafnia alvei* HA4597 bacteria were obtained from EnteroSatys^®^ caps (TargEDys, Longjumeau, France) and isolated on Luria–Bertani (LB) agar. A single colony was incubated in 10 mL of LB broth overnight at 37 °C with orbital shaking (140 rpm). A fraction of resulting culture (0.2 mL) was diluted at 10^7^ CFU/mL in 200 mL (Erlenmeyer flask) of LB broth and incubated at 37 °C with orbital shaking (140 rpm). After a 6 h incubation which corresponded to the beginning of the stationary growth phase which was checked by optical density, the culture was stopped for sample collection. Bacterial abundance was determined by sowing on LB agar; 5 mL of sample contained 3.5 × 10^8^ CFU. Samples were diluted in 0.9% NaCl to a concentration of 4.5 × 10^7^ CFU in 200 µL, a single dose per mouse, similar to that used previously [16]. Aliquots were stored at −20 °C for further use.

### 2.3. Bacterial Protein Extraction

Bacterial protein was extracted according to a previously published protocol [16]. Briefly, samples of *H. alvei* cultures were centrifuged at 3000× *g* for 20 min at 4 °C in Falcon tubes (50 mL) and supernatant was discarded. To wash out secreted compounds, bacterial pellets were resuspended with 0.1 M phosphate-buffered saline (PBS) and centrifuged at 3000× *g* for 20 min at 4 °C, and the supernatant was discarded. Washes were repeated 3 times. To promote lysis, the pellets were frozen overnight at −20 °C. Bacterial cells were lysed in 2 mL R2D2 buffer (7 M urea, 2 M thiourea, 2% CHAPS, 0.05% triphosphobutyl, 20 mM dithiothreitol, 0.5% C7BzO) with sonication for 6 min at 23% amplitude (3 s ON/1 s OFF). The addition of thiourea to the lysis buffer promoted better solubilization of the protein. Solubilized proteins were recovered by taking supernatant after centrifugation at 10,000× *g* for 30 min at 4 °C and quantified using Bradford assay. Protein extracts were diluted in saline (5 µg in 200 µL), a single dose per mouse similar to that used previously [16]. Aliquots were stored at −20 °C for further use.

### 2.4. Experimental Design

Mice were randomly divided into three groups (*n* = 5, in each group) and received the following treatments by gastric gavage once daily for 16 days: the control group received 200 μL of 0.9% NaCl solution/animal; the *H. alvei* protein extract group received 5 µg in 200 µL of saline/animal, and the *H. alvei* suspension group received *H. alvei* live bacteria 4.5 × 10^7^ CFU in 200 µL of saline/animal). All of the animals completed the study without attrition or adverse events.

### 2.5. Body and Fat Weight

The body weight of each mouse was measured daily before drug administration. Fat deposits were removed from various body parts at the end of the experimental period after euthanasia of the animals. Total fat included interscapular and subcutaneous fat (somatic fat), as well as splenic, renal, mesenteric, and perigonadal fat (visceral fat). Visceral fat included the anterior subcutaneous (interscapular), posterior subcutaneous (dorsolumbar, inguinal, and gluteal), visceral perirenal, visceral mesenteric and retroperitoneal visceral epididymal (gonadal) bilateral fat depots.

### 2.6. Analysis of the Activity of Brush Border Intestinal Digestive Enzymes

At the next day after the last gavage procedure, mice were killed by decapitation, and the intestines were removed and divided into five sections: duodenum, proximal and distal jejunum, ileum, and large intestine. To obtain the chyme fraction, each section of the intestine was washed with 5 mL of Ringer’s solution (pH 7.1–7.4). Then, to obtain the mucosal fraction, the mucosa was scraped off and diluted 50 times with Ringer’s solution (pH 7.1–7.4), then both fractions were homogenized. The activities of intestinal enzymes were determined in the obtained homogenates. The activity of glucoamylase (EC 3.2.1.3) and maltase (EC 3.2.1.20) was assessed by the increase in glucose formed during the hydrolysis of starch and maltose, respectively, at substrate concentrations of 20 g/L [17,18]. The activity of alkaline phosphatase (EC 3.1.3.1) was assessed by the increase in p-nitrophenol formed during the hydrolysis of sodium p-nitrophenyl phosphate, 0.6 mM [18]. Activity of aminopeptidase N (EC 3.4.11.2) was measured by the increase in β-naphthylamine using 0.75 mM D, L-alanine-β-naphthylamide as a substrate [19]. The enzymatic activity was expressed in micromoles of the hydrolyzed substrate for 1 min per 1 g of the wet mass of the chyme or mucosal tissue.

### 2.7. Morphological Analysis

For postmortem morphological analysis of the small intestine, tissue samples about 0.5 cm long were taken from two sections (the middle of the jejunum and the middle of the ileum). The samples were washed in phosphate buffer (pH 7.4) and fixed for 24 h in 10% formalin prepared in phosphate buffer (pH 7.4). The samples were then dehydrated in several changes in isopropyl alcohol and embedded in paraffin according to the standard histological technique [20]. Sections (4–5 µm thick) were cut using a Microm HM325 microtome (Thermo Fisher Scientific, Waltham, MA, USA) and stained with hematoxylin–eosin. The sections were analyzed in a Nikon Ni-U light microscope equipped with a DS-Fi2 camera using the NIS-Elements program (BR4.30). The height of the villi (at least 10 sections from each site), the number of enterocytes per longitudinal section of the villus (10 sections from each sample with an interval of 120 μm), as well as the number of mature goblet cells and lymphocytes in the villous epithelium were determined.

### 2.8. Electron Microscopy

For postmortem electron microscopic analysis, colon tissue samples of about 0.5 cm in length were taken from control and *H. alvei*-treated mice. Tissue samples were prepared according to the standard protocol [21]. Briefly, colon fragments were washed in PBS, pH 7.4, fixed with 2% glutaraldehyde in PBS, washed with PBS, post-fixed with 1% OsO_4_ in PBS, washed again with PBS and dehydrated with increasing concentrations of ethyl alcohol and acetone. Then the tissue samples were embedded in Epon (Sigma-Aldrich, St. Louis, MO, USA). Ultrathin sections were prepared from the specimen, including blocks using the Leica EM UC7 ultratome (Leica Microsystems, Mannheim, Germany), then contrasted with uranyl acetate and lead citrate. The sections were studied under an electron microscope Jeol JEM–2100 (JEOL Ltd., Tokyo, Japan).

### 2.9. Statistical Analysis

The results are represented as the mean ± standard error of means (SEM). The normality of data distribution was tested using the Shapiro–Wilk test. The significance of differences between the groups was analyzed using one-way ANOVA and Tukey’s HSD post hoc test and in some cases also Student’s *t*-test, the ANOVA–LSD test, the Kruskal–Wallis test and the Mann–Whitney test (indicated in the text). Linear correlations between variables were calculated by the Pearson’s R correlation test using Origin 7.0 software (Origin Lab Corp., Northampton, MA, USA). Differences were considered to be statistically significant with *p* values < 0.05.

## 3. Results

### 3.1. Body, Feed Consumption, Fat and Chyme Weights

At the end of the experiments, the body weight of mice in all study groups slightly decreased compared to the initial level (before the administration of the drugs or their solvent). Thus, in the control group, this indicator decreased (compared to the initial level) from 28.89 ± 0.28 g to 28.46 ± 0.38 g (*p* = 0.043, nonparametric Mann–Whitney test); in the group receiving *H. alvei* protein, it did not differ significantly from the initial level (initial level—28.04 ± 0.22 g, at the end of the experiments—27.95 ± 0.32), and in the group receiving *H. alvei* suspension, there was a tendency for this indicator to decrease compared to the initial level (initial level—29.00 ± 0.68 g, at the end of the experiments—28.51 ± 0.42, *p* = 0.095, mixed ANOVA test). Differences in feed consumption were noted between the study groups during the experiments. Thus, in the *H. alvei* protein group, the average feed consumption rate (over 16 days of the experiment) per gram of body weight was 0.528 ± 0.021 kcal/day/g, which is 8.4% higher (*p* < 0.01, Student’s *t*-test) than that of the control group (0.487 ± 0.052 kcal/day/g). At the same time, in the *H. alvei* suspension group this indicator was 0.463 ± 0.018 kcal/day/g and it was only slightly lower than in the control group (*p* > 0.05, Student’s *t*-test), but was 12.3% lower than in the *H. alvei* protein group (*p* < 0.05, Student’s *t*-test).

The total mass of fat extracted from various body depots (interscapular, subcutaneous, mesenteric and perigonadal fats) in the *H. alvei* protein group was 1.07 ± 0.14 g, which was no different from that in the control group (1.071 ± 0.051 g), and in the group receiving *H. alvei* suspension, fat mass (0.90 ± 0.11 g) was only slightly lower (*p* > 0.05, Student’s *t*-test) than in the control group and the group receiving *H. alvei* protein.

**Figure 1:**
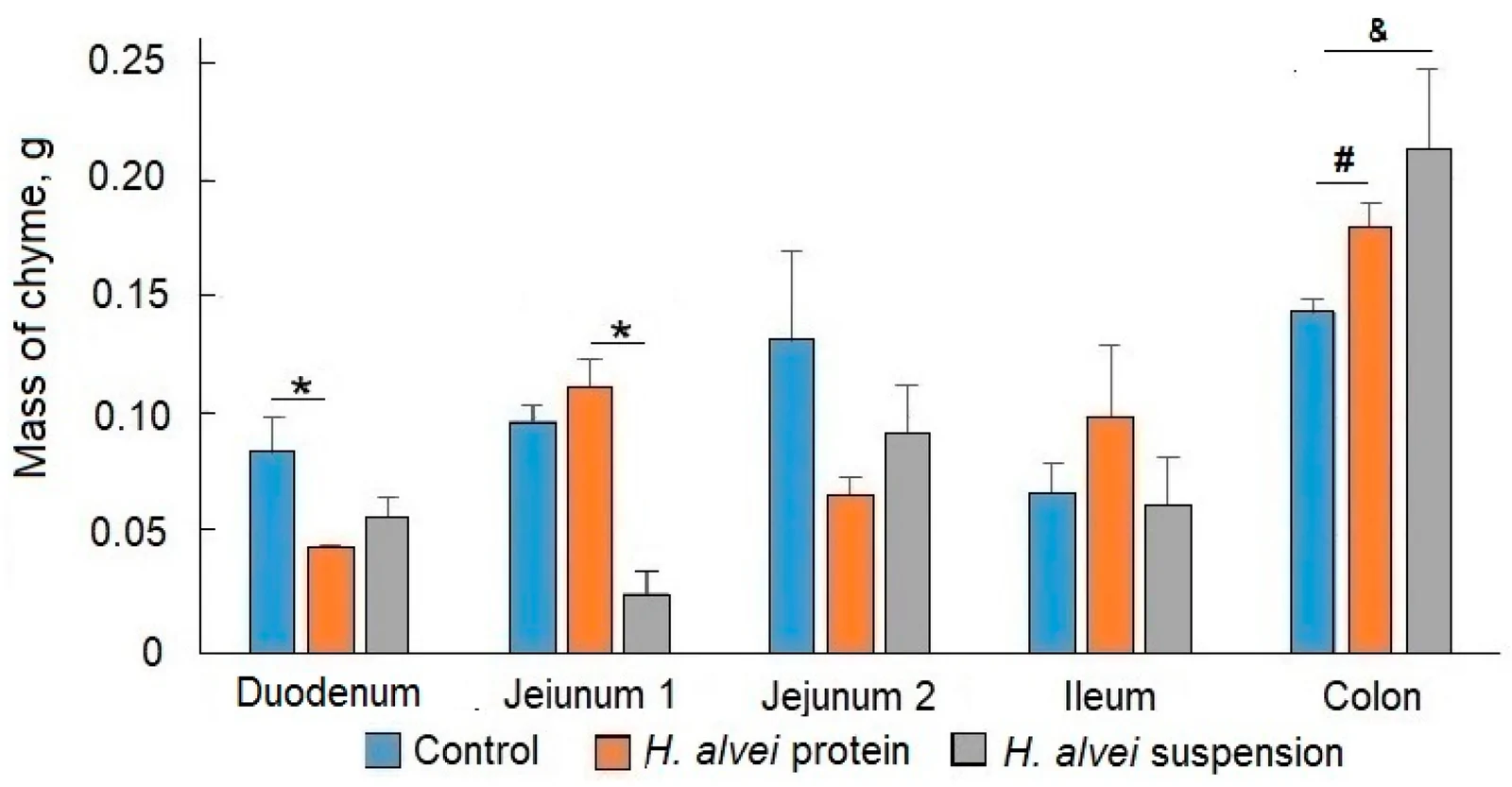
The effect of 16-day administration of *H. alvei* protein and *H. alvei* suspension on the chyme mass in different parts of the mouse intestine. Jejunum 1—proximal jejunum; Jejunum 2—distal jejunum. One-way ANOVA and Tukey’s HSD post hoc test, * *p* < 0.05; Kruskal–Wallis test, # *p* = 0.027; ANOVA-LSD test, ^&^ *p* = 0.034.

The chyme mass in mice was reduced by 48% (*p* < 0.05) in the duodenum in the *H. alvei* protein group compared to the control group, and by 58% (*p* < 0.05) in the proximal jejunum in the *H. alvei* suspension group compared to the *H. alvei* protein group (Figure 1). However, the colonic chyme mass in mice of the *H. alvei* protein group increased by 25% (*p* = 0.027, Kruskal–Wallis test) while that of the *H. alvei* suspension group increased by 48% (*p* = 0.034, ANOVA-LSD test) compared to the control group (Figure 1).

### 3.2. Morphological Analysis of the Jejunum and Ileum

**Figure 2:**
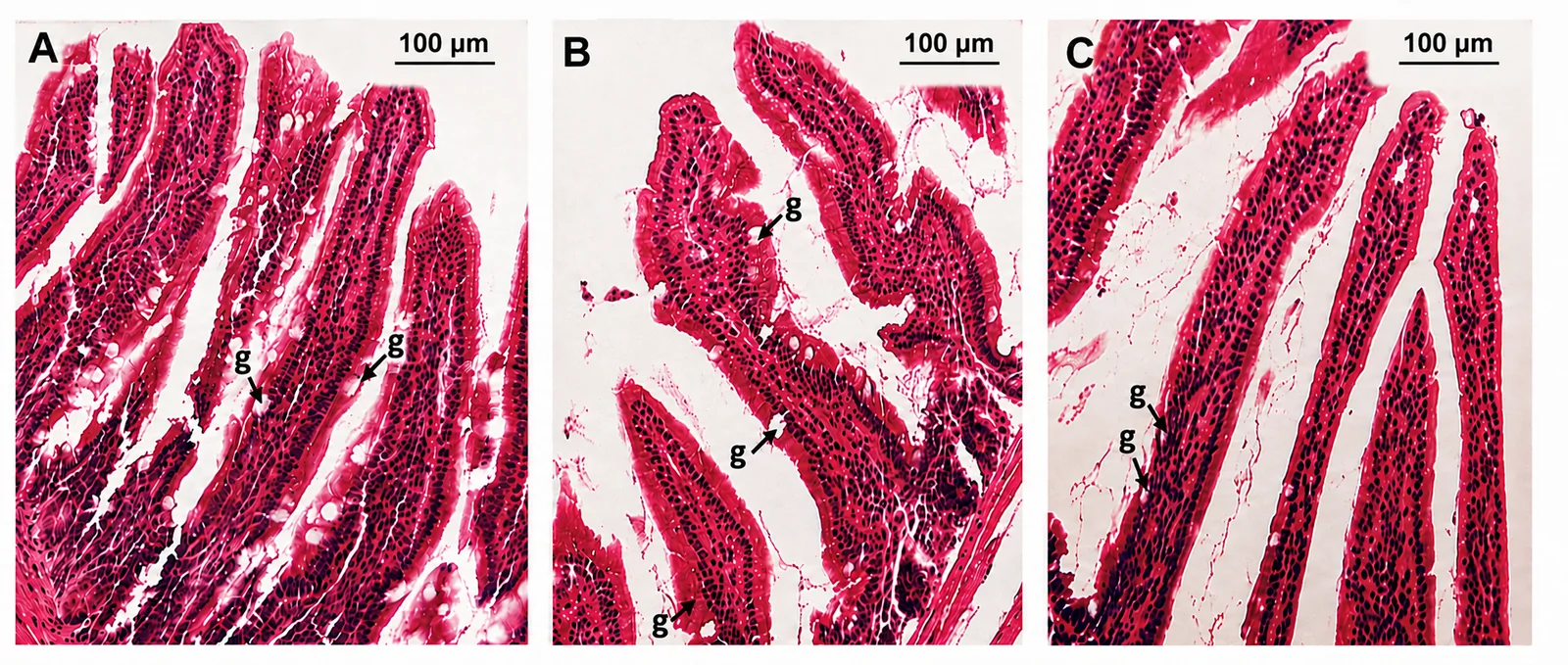
Histological sections of the mouse jejunum. Goblet cells (g) indicated by the arrows: (**A**)—control group; (**B**)—*H. alvei* protein group; (**C**)—*H. alvei* suspension group. Scale bar (**A**–**C**)—100 µm.

**Figure 3:**
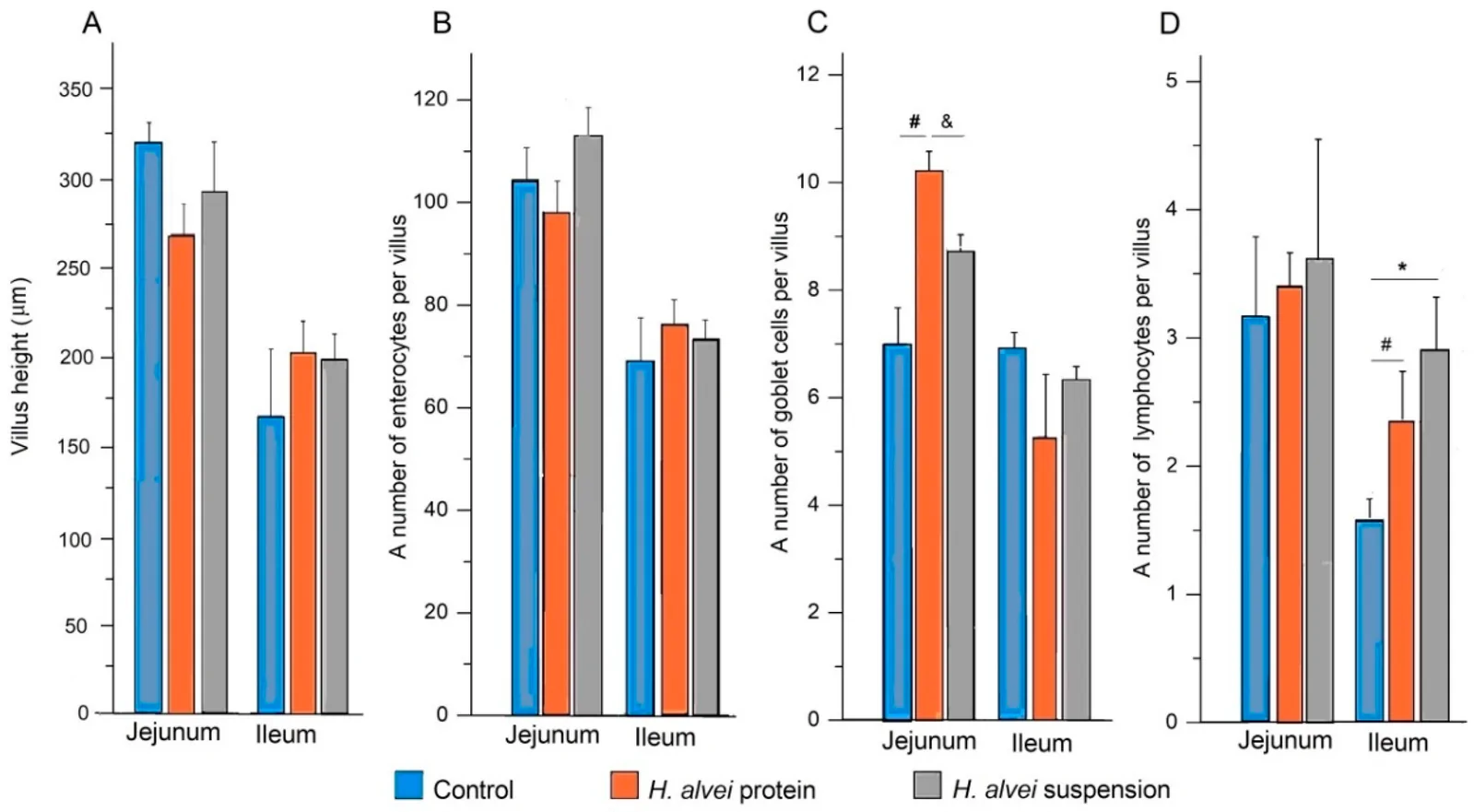
The effect of 16-day administration of *H. alvei* protein and *H. alvei* suspension on the structural characteristics of the jejunum and ileum in mice. (**A**) The height of the villi; (**B**) the number of enterocytes in the villi; (**C**) the number of goblet cells in the epithelium of the villi; (**D**) the number of lymphoid cells in the epithelium of the villi. (**C**) ANOVA-LSD test, ^#^ *p* = 0.093, ^&^ *p* = 0.077. (**D**) Kruskal–Wallis test, * *p* = 0.05; Mann–Whitney test, ^#^ *p* = 0.1.

Goblet cells were present in the intestinal villi of all study groups (Figure 2A–C). In the group receiving *H. alvei* protein, the number of goblet cells in the jejunal villi tended to increase by 31% (*p* = 0.093, ANOVA-LSD test) compared with the control group and by 22% (*p* = 0.077, Kruskal–Wallis test) compared with the group receiving *H. alvei* suspension (Figure 3C).

**Figure 4:**
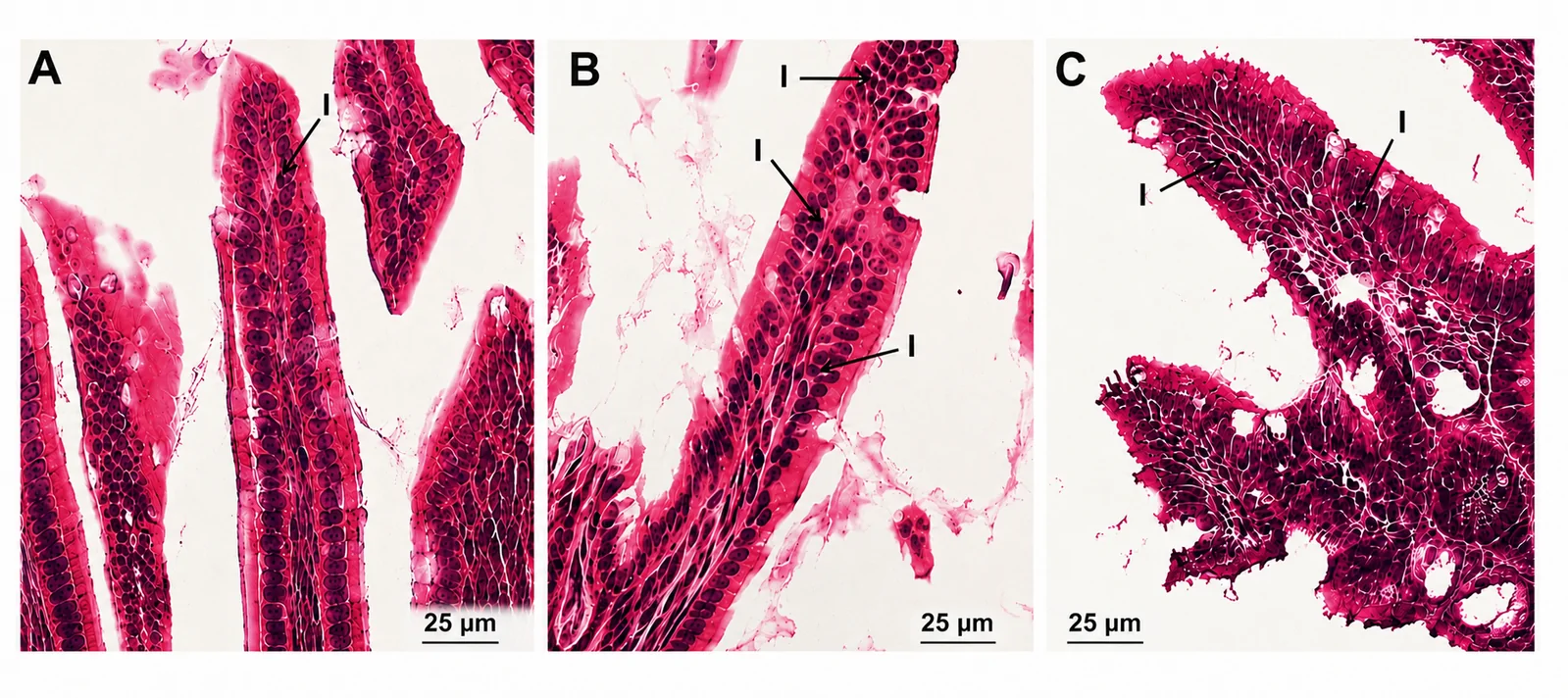
Histological sections of the mouse ileum. Lymphoid cells (l) in the villus are indicated by arrows: (**A**) control group; (**B**) *H. alvei* protein group; (**C**) *H. alvei* suspension group. Scale bar (**A**–**C**)—25 µm.

Regarding the intraepithelial lymphocytes on the intestinal villi, their number in the jejunum in both groups (*H. alvei* protein and *H. alvei* suspension) was no different from that in the control group (Figure 3D). However, in the group receiving *H. alvei* protein, this indicator tended to increase (compared to the control group) in the ileum (*p* = 0.1, Mann–Whitney test), and in the group receiving *H. alvei* suspension, it increased by 2.3 times (*p* = 0.05, Kruskal–Wallis test) (Figure 3D and Figure 4A–C).

**Figure 5:**
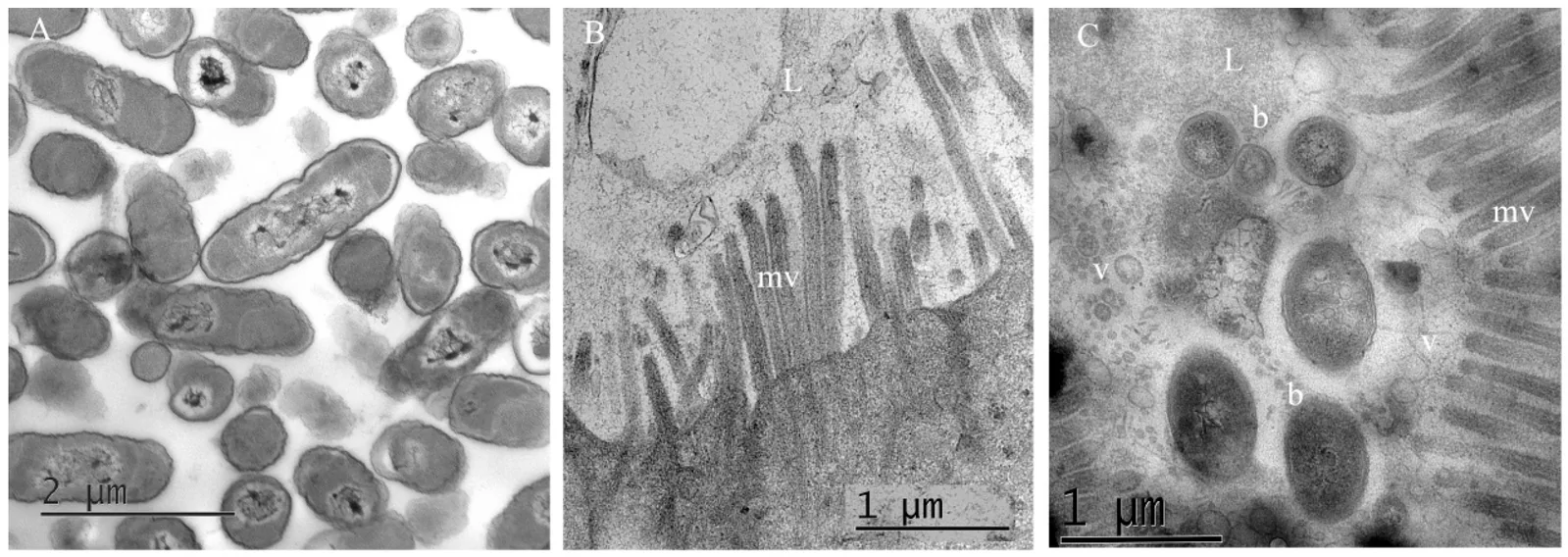
Electron micrographs of *H. alvei* bacterial cells cultured in vitro (**A**), sections of colonocytes of mice in control group (**B**) and after administration of *H. alvei* suspension (**C**). In electron micrographs (**B**,**C**): b—bacteria, L—lumen of the colon, mv—microvilli, v—secretory vesicles. Scale bar (**A**)—2 µm; (**B**,**C**)—1 µm.

The height of the villi and the number of enterocytes on the villi of the jejunum and ileum in mice of both groups (*H. alvei* protein and *H. alvei* suspension) did not differ significantly from similar indicators in the control group (Figure 5A,B).

### 3.3. Electron Microscopic Analysis

*H. alvei* cultured in vitro (Figure 5A), as well as microvilli on the apical surface of colonocytes from a control mouse (Figure 5B) and a mouse receiving *H. alvei* suspension (Figure 5C) were analyzed using electron microscopy. In control mice, bacteria were difficult to detect near the microvilli of colonocytes (Figure 5B). After administration of a suspension of *H. alvei* to mice, numerous *H. alvei*-like bacteria surrounded by secretory vesicles were found in the intestinal lumen near the microvilli of colonocytes (Figure 5C).

### 3.4. Enzymatic Activity in the Intestinal Mucosa

**Figure 6:**
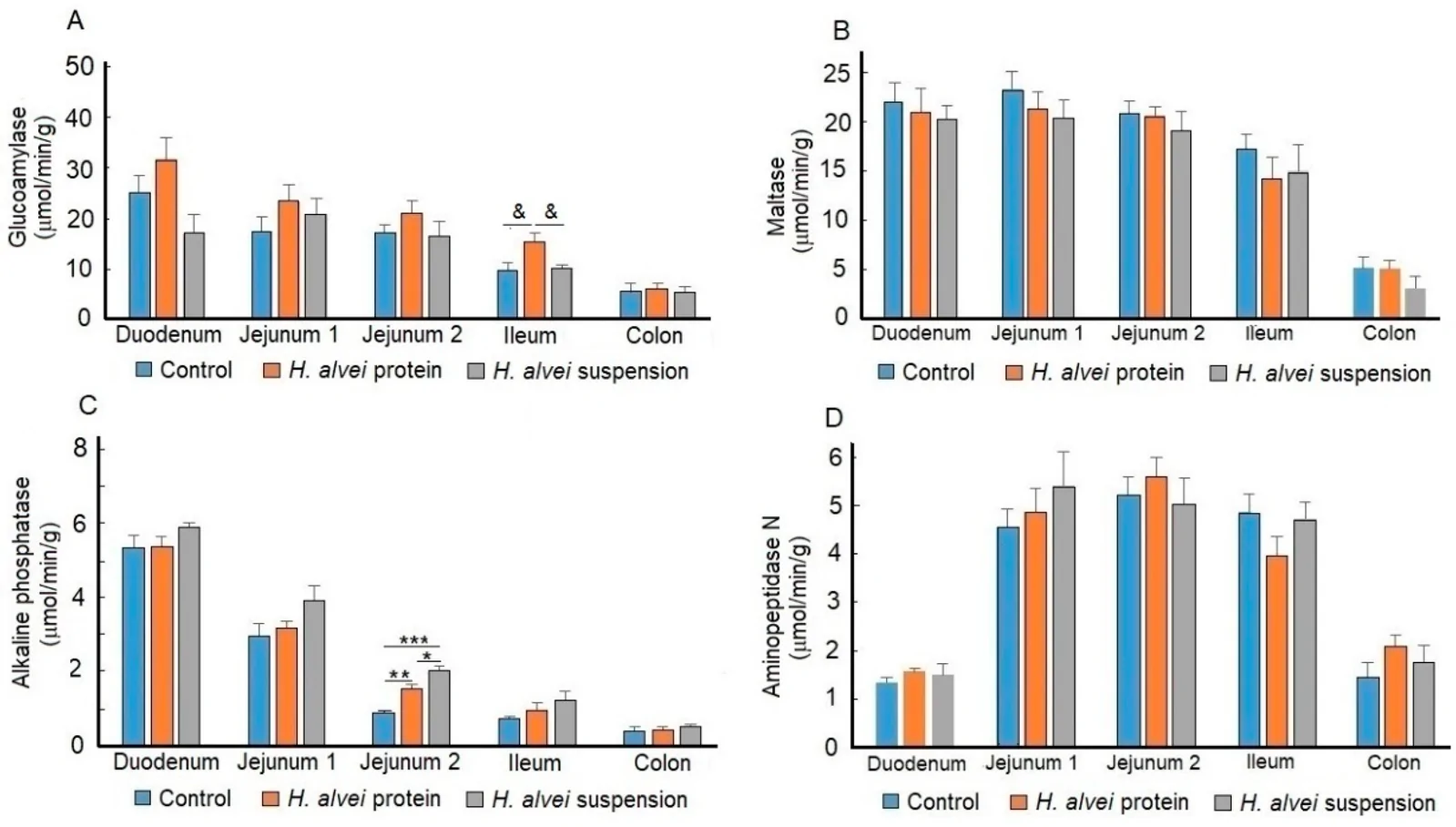
The effect of 16-day administration of *H. alvei* protein and *H. alvei* suspension on enzyme activity in the mucosa of various parts of the mouse intestine. (**A**) Glucoamylase activity; (**B**) maltase activity; (**C**) alkaline phosphatase (ALP) activity; (**D**) aminopeptidase N (APN) activity. Jejunum 1—proximal jejunum; Jejunum 2—distal jejunum. (**A**) ANOVA-LSD test, ^&^ *p* = 0.022; (**C**) one-way ANOVA and Tukey’s HSD post hoc test, * *p* < 0.05, ** *p* < 0.01, *** *p* < 0.0003.

Introduction of *H. alvei* protein resulted in an increase in glucoamylase activity in the ileal mucosa by 60% (*p* = 0.022, ANOVA-LSD test) compared with the control group and the *H. alvei* suspension group by 50.7% (*p* = 0.048, ANOVA-LSD test) (Figure 6A). However, after introduction of *H. alvei* suspension, the activity of this enzyme in the mucosa of various parts of the intestine did not differ from that in the control group.

The introduction of both preparations (*H. alvei* protein, *H. alvei* suspension) did not affect the activity of maltase in the mucosa of various parts of the intestine (Figure 6B).

Under the influence of both preparations (*H. alvei* protein, *H. alvei* suspension), the alkaline phosphatase (ALP) activity in the mucosa of the distal jejunum increased compared with the control group (in the *H. alvei* protein group it increased by 62%, *p* < 0.01, while in the *H. alvei* suspension group it increased by 2.2 times, *p* < 0.0003) (Figure 6C). Notably, in the *H. alvei* suspension group, the ALP activity level in this part of the intestine was 36% higher (*p* < 0.05) than in the *H. alvei* protein group.

Neither preparation (*H. alvei* protein or *H. alvei* suspension) affected the aminopeptidase N (APN) activity in the mucosa of various parts of the intestine (Figure 6D).

### 3.5. ALP Activity Correlates with the Amount of Fat Tissue

**Figure 7:**
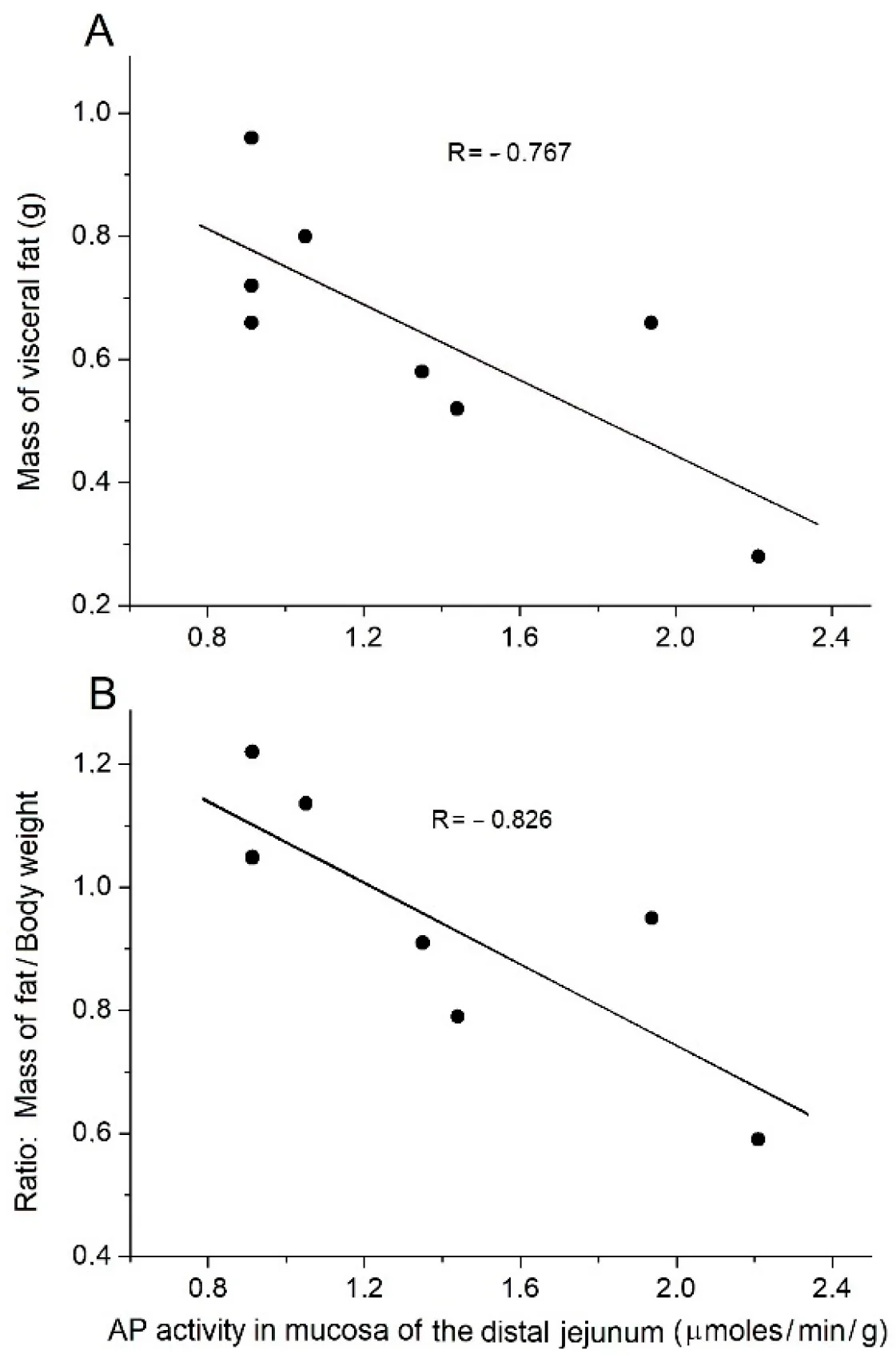
Relationship between ALP activities in the mucosa of the distal jejunum and visceral fat mass (**A**) or ratio: total fat mass/body weight (**B**). (**A**) Pearson’s R test, *p* = 0.026, *n* = 8. (**B**) Pearson’s R test, *p* = 0.01, *n* = 8.

Given the fact that ALP in the small intestinal mucosa is involved in fat absorption [12,13,22,23,24], we analyzed the relationship between ALP activity in the mucosa of the distal jejunum and visceral fat mass in the body or ratio total fat mass/body weight using data from all groups of mice. The results showed a negative linear correlation between the analyzed parameters: in the case of visceral fat (visceral perirenal, visceral mesenteric, and retroperitoneal visceral epididymal (gonadal) bilateral fat depots), the correlation coefficient R = −0.767, *p* = 0.026, while in the case of total fat (interscapular, subcutaneous, mesenteric and perigonadal fats), R = −0.826, *p* = 0.011 (Pearson’s R correlation test) (Figure 7).

### 3.6. ALP Activity in Chyme

**Figure 8:**
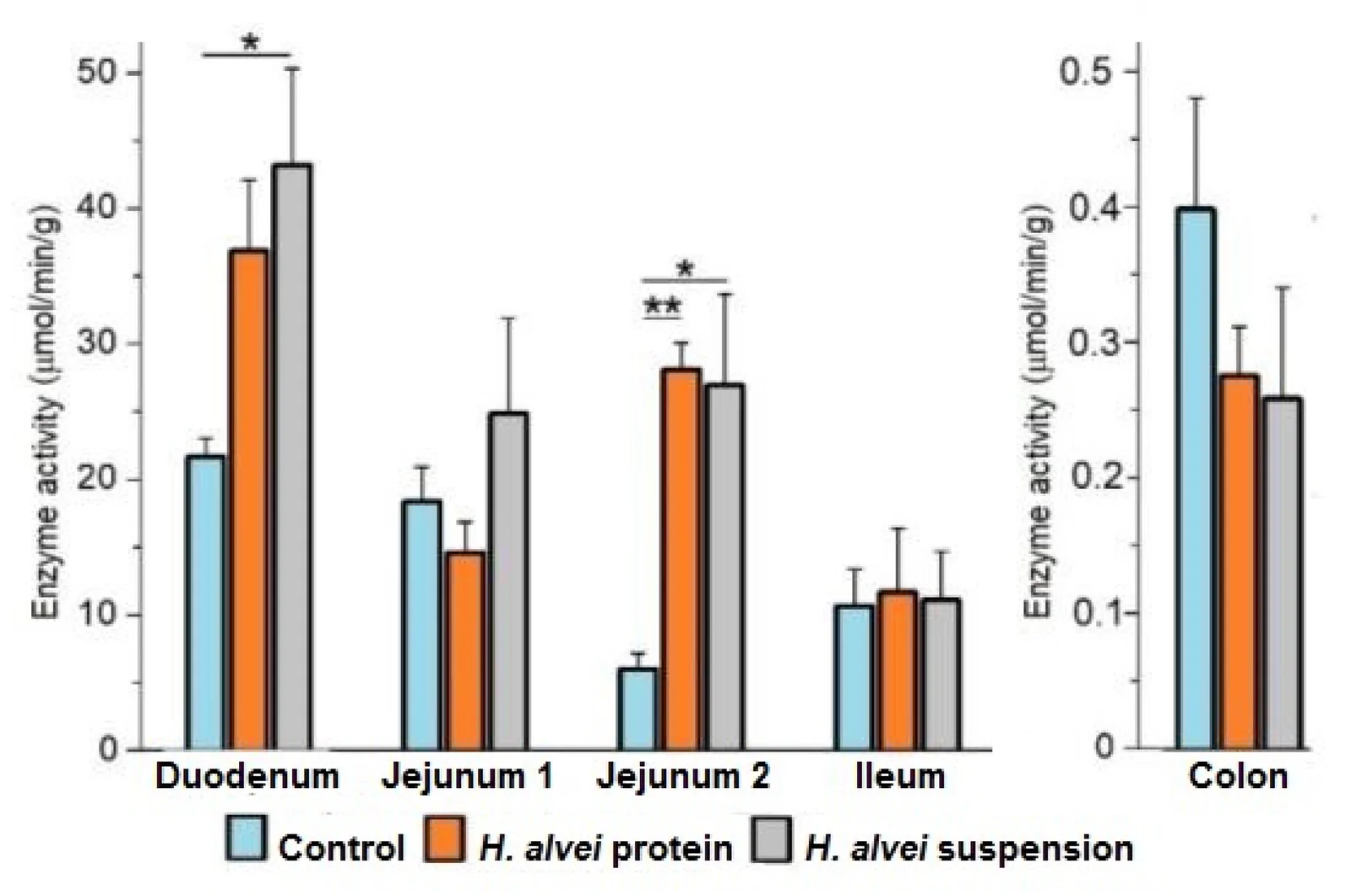
The effect of 16-day administration of *H. alvei* protein and *H. alvei* suspension on alkaline phosphatase (ALP) activity in the chyme of different parts of the mouse intestine. Jejunum 1—proximal jejunum; Jejunum 2—distal jejunum. One-way ANOVA and Tukey’s HSD post hoc test, * *p* < 0.05, ** *p* < 0.003.

Both preparations significantly increased the activity of ALP compared to control in the intestinal chyme of the distal jejunum: 4.0 times (*p* < 0.05) and 4.1 times (*p* < 0.017) for the *H. alvei* suspension and *H. alvei* protein, respectively. In the duodenum, the *H. alvei* suspension increased the activity of ALP compared to control in the chyme by 2.0 times (*p* < 0.05) whereas the *H. alvei* protein group increased the activity of ALP compared to control less significantly, by 71% (*p* = 0.068, ANOVA-LSD test) (Figure 8).

## 4. Discussion

Digestive enzymes of the brush border membrane of enterocytes play a vital role in digestion. By mediating the intermediate and final stages of digestion, they can thus significantly influence the rate of absorption of the resulting substances into the body’s internal environment [25]. It is also important to note that intestinal microbiota has a significant impact on its structure (intestinal morphology, number of goblet cells and lymphocytes) and its functional characteristics [24,25,26].

We report here that 16-day administration of *H. alvei* protein extract to mice increases the number of goblet cells in the villi of the jejunum, which indicates an increase in mucus production and points to the activation of this non-specific defense mechanism. At the same time, when *H. alvei* protein or *H. alvei* suspension were administered, an increase in the number of intraepithelial lymphocytes on the villi of the ileum was observed, which indicated the activation of a specific immune defense mechanism.

Our study also revealed that administration of *H. alvei* preparations increases the activity of intestinal alkaline phosphatase (ALP). In addition to participating in hydrolysis of food substrates, ALP plays a substantial role in maintaining intestinal homeostasis making it an attractive drug candidate for several gastrointestinal-related disorders [24,27,28]. Indeed, intestinal ALP supports pH in the duodenum, detoxifies bacterial toxins (lipopolysaccharide [LPS] and flagellin), and is also involved in fat absorption and in intestinal microbiota control [13,14,15,16]. In addition to its protective local effect, intestinal ALP improves brain function and behavior and prevents aging [9,29,30].

In this study, we demonstrated that both preparations (*H. alvei* protein and *H. alvei* suspension) significantly increased the ALP activity in jejunal mucosa (×1.5 and ×2.2 times, respectively), as well as in chyme of the duodenum (×1.7 and ×2.0 times, respectively) and the jejunum (×4.7 and ×4.5 times, respectively). Since *H. alvei* belongs to Gram-negative bacteria containing LPS in their wall, it is reasonable to expect such a reaction of ALP in response to an increase in the level of LPS in the lumen of the small intestine in the *H. alvei* suspension group of mice. However, the *H. alvei* protein preparation does not contain membranes of the *H. alvei* bacteria, and therefore, the observed increase in ALP activity in the intestinal mucosa and chyme in mice of the group *H. alvei* protein is apparently LPS-independent but can be due to the presence of specific regulatory proteins. Indeed, a small daily dose of *H. alvei* protein extract (5.0 µg) is about one million time lower than the daily food intake of a healthy mouse, i.e., this bacterial extract cannot possibly have any nutritional effect. We may speculate that bacterial ClpB protein could be the active substance underlying the increase in the intestinal ALP activity via activation of melanocortin receptor type 4 (MC4R) in the gut. Indeed, both Enterobacterial ClpB protein and *H. alvei* protein extract were shown to activate MC4R [31,32].

Importantly, expression of MC4R was detected in enterocytes, where it modulates lipid absorption [32], suggesting a direct regulatory effect of *H. alvei* proteins on enterocyte function. An earlier study showed that administration of adrenocorticotropin, i.e., a melanocortin peptide, to rats leads to a rise in intestinal alkaline phosphatase levels [33]. Moreover, indirect pathways can also be involved, via enteroendocrine L-cells known to express MC4R in both rodents and humans and whose activation stimulates secretion of glucagon-like peptide 1 (GLP-1) and peptide tyrosine–tyrosine (PYY) [34,35]. ClpB at nanomolar concentrations activated PYY secretion of intestinal cell cultures [36], while PYY was shown to directly activate intestinal ALP activity [37].

It is noticeable that in our experiments, ALP activity did not increase in the chyme of the lower intestine, while the electron microscopy data showed the presence of bacteria similar to *H. alvei* near the apical membrane of colonic enterocytes. This discrepancy can apparently be explained by the fact that the amount of ALP, which is produced in the epithelium of duodenum and in the intestinal lumen, was insufficient to reach lower gut compartments. It does not, however, exclude the possibility that some metabolites such as ClpB can be released from *H. alvei* bacteria in the lower gut and can act systemically, including via stimulation of PYY secretion [8,23].

The ALP in the jejunal mucosa is known to limit fat absorption [13,14,22,27,38]. This property may underlie our finding of negative correlations between the levels of ALP activity in the jejunal mucosa and both total fat mass and visceral fat even under conditions of mice fed a standard diet. This finding indicates that these properties of *H. alvei* may contribute to its previously established anti-obesity effects mediated by bacterial ClpB protein-activating MC4R.

It is also interesting to note that in this study we observed an increase in glucoamylase activity in the ileal mucosa of mice after administration of *H. alvei* protein extract. This contrasts with the low activity of this enzyme in the small intestine of rats with type 2 diabetes observed in our previous study [39]. In this regard, one can consider the potential of using *H. alvei* preparations to restore glucoamylase activity in patients with type 2 diabetes aiming to increase glucose utilization in the gut, in addition to *H. alvei*’s efficient glucose consumption [40].

It is noteworthy that neither of the *H. alvei* preparations affected the activity of another important intestinal brush border enzyme, APN, which, in addition to being involved in protein digestion, is also involved in cholesterol transport, immune responses, and antigen presentation, and is a receptor for viruses [11,12]. Thus, the use of *H. alvei* preparations should not affect protein digestion, cholesterol transport or other adverse processes associated with the function of the APN.

Finally, under the influence of both *H. alvei* preparations, a decrease in the mass of chyme in the upper intestine and its increase in the colon were observed. Considering that changes in the mass of chyme along the intestine are closely related to changes in its motility, it can be assumed that the probiotic *H. alvei* has a stimulating effect on intestinal motility in the upper sections. In this context, MC4R was shown to be expressed in the vagal afferents [23], i.e., it can mediate post-meal intestinal reflexes [41]. In contrast, in the lower sections of the intestine that are rich in enteroendocrine L-cells, MC4R-mediated PYY release can slow down the intestinal transit, as reflected by increased chyme [42].

## 5. Conclusions

Our data show that the gut reacts to the administration of both suspension of live *H. alvei* bacteria and their total protein extract in a similar way, as reflected by increased activity of the brush border ALP enzyme in both jejunal mucosa and in the chyme of the duodenum and jejunum. At the same time, other intestinal protective mechanisms are activated, including a higher number of mucus-producing cells in the jejunum and interepithelial lymphocytes in the ileum. Taken together, *H. alvei* preparations display beneficial protective effects on gut health.

## 6. Limitations

The findings are derived from an animal model and should therefore be interpreted within this experimental context and a relatively small sample, which may affect the interpretation and generalization of the results.

## Data Availability

The data presented in this study are available on request from the corresponding authors.
